# Development of Protein‐Source and Fat‐Free Indonesian Traditional Crackers (*kerupuk*) Through Egg White Substitution and Alternative Frying

**DOI:** 10.1002/fsn3.71208

**Published:** 2026-02-24

**Authors:** Ata Aditya Wardana, Kaneth, R. Haryo Bimo Setiarto, Retno Wulandari, Laras Putri Wigati, Fumina Tanaka, Fumihiko Tanaka

**Affiliations:** ^1^ Food Technology Department, Faculty of Engineering Bina Nusantara University Jakarta Indonesia; ^2^ Research Center for Applied Microbiology, National Research and Innovation Agency (BRIN) Bogor West Java Indonesia; ^3^ Research Center for Polymer Technology, National Agency for Research and Innovation South Tangerang Indonesia; ^4^ Laboratory of Postharvest Science, Faculty of Agriculture Kyushu University Fukuoka Japan

**Keywords:** functional, health, nutrition, processing, traditional snacks

## Abstract

Commercially available *kerupuk* (Indonesian traditional crackers) are typically high in fat and low in protein, making them nutritionally imbalanced and categorized as junk food. This study aims to develop a protein‐source and fat free traditional crackers through egg white powder (EWP) substitution and application of alternative frying methods. The substitution of EWP in was selected at a 20% level based on preliminary test ranging from 0% to 40% and the alternative frying methods were deep frying (control), air frying, sand frying, and microwaving. The developed traditional crackers was characterized with pH value between 7.20 and 7.34; water activity (*a*_w_) between 0.41 and 0.42; protein and fat levels respectively ranging from 18.26% to 18.73% and 0.19%–0.41%, meeting the claims as a protein source and fat free product according to Indonesian Food and Drug Authority (BPOM). SEM analysis showed that microwave cooking and deep‐frying produced traditional crackers with highly porous structures with large pores. FTIR analysis indicated that deep‐fried traditional crackers showed intense peaks at 2930–2850 cm^−1^ (C–H stretching) and 1743 cm^−1^ (C = O ester carbonyl stretching), indicating high oil absorption, whereas alternative frying methods showed lower intensities of these lipid‐associated peaks. Traditional crackers produced had a pH value ranging from 7.20 to 7.34 and water activity (*a*_w_) between 0.41 and 0.42. Microwave emerged as the optimal alternative frying technique, with the highest expansion rate (185.63%), crispness (37.58 mm), and brightness (L* = 92.88).

## Graphical abstract

The substitution of EWP was selected at a 20% level based on preliminary test ranging from 0% to 40% and the alternative frying methods were deep frying (control), air frying, sand frying, and microwaving. The developed kerupuk was characterized with pH value between 7.20 and 7.34; water activity (*a*_w_) between 0.41 and 0.42; protein and fat levels respectively ranging from 18.26% to 18.73% and 0.19%–0.41%, meeting the claims as a protein source and fat free product according to Indonesian Food and Drug Authority (BPOM).

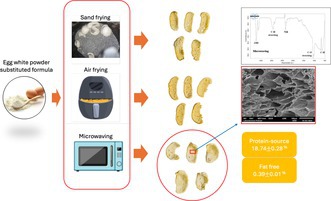

## 1 Introduction

Crackers, locally known in Indonesia as *kerupuk*, have been deeply rooted in Indonesian culinary heritage, with historical references found in ancient Javanese manuscripts (De Bie 1901; De Kruijff 1909; Santoso 1980). This is confirmed by photographic documentation of cracker sales activities in several traditional markets across Java Island, which are preserved in various museums and libraries (Wereldmuseum 1910, 1932). After Indonesia's independence in 1945, cracker‐eating contests were introduced as part of national celebrations, reflecting both communal joy and remembrance of past hardships (Finaka 2022).

This type of crackers not only offers a distinctive taste and culture, but also reflects the rich culinary diversity of the country. Typically served as a side dish or enjoyed as a standalone snack, crackers are favored for their crispy texture, savory flavor, and relatively low cost (Asikin et al. 2019). They are generally made from starch‐based ingredients, such as tapioca flour, combined with spices and salt. A wide variety of crackers exists, depending on the raw materials used, including onion, fish, shrimp, and animal skin (Cahyono and Nurcahyo 2022). However, excessive consumption of conventional crackers may pose health risks, as most commercially available products are high in calories and fat but low in protein. This concern is primarily attributed to the traditional deep‐frying process. During frying, high temperatures cause water to evaporate, resulting in substantial oil absorption and increased fat content in the final product. *Crackers* absorb approximately 14%–30% of oil during frying (Zulfahmi et al. 2021). Supporting evidence demonstrated that deep‐frying significantly increases fat content in crackers, with studies showing fat levels raised from 4.32% to 7.72% in prawn crackers and 3.13% to 6.72% in milk crackers (Susanti and Wibawa 2023). Elevated fat content is often linked to metabolic disorders, including cardiovascular disease, obesity, and hypercholesterolemia (Thaha et al. 2018).

In Indonesia, undernutrition and malnutrition remain major public health concerns. The prevalence of severe malnutrition is 3.9%, while that of undernutrition is 13.8% (Riskesdas 2018). Protein‐energy malnutrition is among the key nutritional problems, with a prevalence of 17.7% based on weight‐for‐age measurements (Riskesdas 2018). In addition, fat intake among the Indonesian population is relatively high, with an average consumption of 53.3 g per capita per day. Notably, 27% of the population consumes more than 67 g of fat daily. In response to these health concerns, crackers present a potential opportunity for nutritional intervention due to their high consumption rate. According to data from Statistics Indonesia (BPS 2024), the average weekly consumption of crackers across the country ranges from 0.13 to 0.15 kg per person, a figure that surpasses the consumption of other snack foods.

Until now, only limited studies have been found reporting strategies to improve the nutritional quality of crackers. The use of Spanish mackerel in cracker formulation with the deep frying method has yielded products with better protein contents ranging from 12.88% to 20.71%, fat contents between 1.27% and 3.01%, and carbohydrate levels of 71.17%–76.95% (Zulfahmi et al. 2014). Another study demonstrated that substituting 10% of the base flour with *rebon* shrimp flour and processing it with the deep frying method resulted in crackers with an expansion ratio of 260.67%, a fat content of 0.95%, and a protein content of 9.03% (Multazam et al. 2023). However, those studies were still unsatisfactory, as they did not meet the requirements for fat‐free and source‐of‐protein claims based on the Indonesian Food and Drug Authority (BPOM), which required less than 0.5 g of fat per 100 g (in solid form) for a fat‐free claim and at least 20% of the nutrient reference value (NRV) per 100 g (in solid form) for a source‐of‐protein claim (BPOM 2022).

No research has focused on the use of egg white powder (EWP) as a functional ingredient in crackers. The EWP is a high‐quality protein source, containing over 80% protein and a complete amino acid profile (Zhu et al. 2018). Its inclusion has been shown to enhance nutritional value without compromising sensory acceptance (Ekafitri et al. 2016). In addition, EWP has versatile applications in the food industry, serving as a protein fortificant in baked goods, meat products, and extruded snacks, making it an important ingredient to help address the increasing demand for nutritious egg‐based products (Hidayat et al. 2024). Thus, in this work, EWP was considered to have promising potential as a protein‐enriching ingredient in cracker production. Furthermore, alternative frying techniques have been explored to develop healthier crackers. Microwave cooking for 60 s has been reported to yield crackers with low fat content (0.25%–0.44%) and expansion volumes of 153%–198% (Rosiani et al. 2015). Similarly, sand frying has produced crackers with expansion volumes ranging from 459% to 725% (Suryaningrum et al. 2016). Furthermore, air frying has demonstrated the ability to significantly reduce fat content while maintaining comparable sensory qualities to deep frying (Cao et al. 2020). Both microwave cooking and air frying rely on rapid heat transfer, enabling faster and more uniform cooking than conventional methods. Fundamentally, each technique minimizes heat gradients to shorten processing time.

Those types of alternative methods offer promising approaches to producing healthier crackers without compromising sensory appeal. However, no research has examined the effects of different frying techniques on the quality characteristics of protein‐source crackers. Hence, this study aims to develop protein‐source and fat‐free crackers through EWP flour substitution and the application of alternative frying methods.

## 2 Materials and Methods

### 2.1 Selection of EWP Level

The best cracker samples were selected based on the protein content, color acceptability, and desirable texture. The selected raw crackers were divided according to the previous section. The combination of tapioca (Pak Tani Gunung, Indonesia) and wheat flour (Telorku, Indonesia) was partially substituted with EWP at concentrations of 0% (control), 10%, 20%, 30%, and 40%. The concentrations of 0%–40% were selected based on preliminary tests, as they were assumed to achieve an optimal balance between nutritional enhancement and sensory acceptability, whereas higher levels resulted in a sandy texture and deviated from typical cracker characteristics. In each formulation, EWP substituted both tapioca and wheat flour proportionally, maintaining the original 4:1 ratio of tapioca to wheat flour in the remaining four blends. This concentration selection was supported by previous research, which indicated that EWP concentration within this range can improve nutritional quality while maintaining acceptable sensory qualities in starch‐based products (Budiman et al. 2009).

The main ingredients were thoroughly mixed with salt, white pepper, shallots, and garlic, according to the formulation in Table 1. Water was gradually added during kneading until a non‐sticky dough was formed. The dough was then shaped into cylindrical forms (5 cm diameter) and steamed at 110°C for 20 min. Subsequently, it was cooled at 4°C for 3 h and sliced into thin pieces (±2 mm). The slices were dried in an oven at 60°C for 6 h. Finally, the dried crackers were fried using a deep‐frying method and subjected to further analysis.

**TABLE 1:** Formulation of crackers.

Ingredients	EWP 0% (g)	EWP 10% (g)	EWP 20% (g)	EWP 30% (g)	EWP 40% (g)
Tapioca	80	72	64	56	48
Wheat flour	20	18	16	14	12
EWP	0	10	20	30	40
Water	75	75	75	75	75
Salt	3	3	3	3	3
White pepper	1	1	1	1	1
Garlic	3	3	3	3	3
Shallot	3	3	3	3	3

### 2.2 Sampel Preparation

Selected crackers were prepared according to the previous section with EVP 20% formulation (Table 1, bold letters). This formulation was selected based on the results presented in Table 2, considering the result of enhanced protein content, color acceptability, and desirable texture observed in the 20% EWP substitution. The raw crackers were divided into four groups according to the frying method: (1) deep frying (control) at 180°C for 30 s; (2) air frying (Lock & Lock, Indonesia) at 180°C for 2 min; (3) sand frying at 190°C for 30 s; and (4) microwave heating (Samsung, Indonesia) at 600 W for 1 min.

**TABLE 2:** Characteristics of the EWP‐substituted crackers.

EWP (%)	Protein content (%)	L*	a*	b*	Hardness (N)
Control	1.92 ± 0.04^a^	63.25 ± 1.78^a^	1.98 ± 0.35^a^	3.99 ± 1.18^a^	3.67 ± 1.42^a^
10	7.70 ± 0.20^b^	69.22 ± 0.94^b^	6.73 ± 1.10^c^	24.42 ± 0.69^c^	6.78 ± 0.50^a^
20	12.90 ± 0.59^c^	69.88 ± 2.70^b^	6.19 ± 0.75^bc^	22.76 ± 1.32^bc^	11.90 ± 0.96^b^
30	16.57 ± 2.03^d^	73.69 ± 1.84^c^	6.10 ± 1.27^bc^	21.89 ± 1.36^b^	14.15 ± 1.41^bc^
40	22.62 ± 0.46^e^	79.49 ± 1.29^d^	5.09 ± 0.55^b^	20.98 ± 1.58^b^	15.93 ± 1.31^c^

*Note:* Different superscript letters indicate significant differences at a 5% significance level (*p* < 0.05).

### 2.3 Color

The color attributes were quantitatively measured using a portable colorimeter (PCS‐CSM 6, Germany) based on the CIE‐L*a*b* color system, defining color in three‐dimensional space using three parameters including L* (lightness), a* (redness/greenness), and b* (yellowness/blueness). Prior to measurement, the colorimeter was calibrated using a standard white calibration plate. The cracker samples were prepared by placing them flat on the calibration plate. The total color difference (Δ*E*) between the sample and the control (deep‐fried crackers) was calculated using the following formula:

$$∆E=\sqrt{{\left(L^{*}-L\right)}^{2}+{\left(a^{*}-a\right)}^{2}+{\left(b^{*}-b\right)}^{2}}$$

where $L^{*}$, $a^{*}$, $b^{*}$ represent the values of the control sample (deep‐fried crackers).

### 2.4 Texture Profile

The texture analysis was performed using a Texture Analyzer (SHIMADZU SM‐168, Japan) with compression level of 20 N. The samples were placed on the instrument's base and compressed with a cylindrical aluminum probe (75 mm diameter) at a velocity of 1 mm/s to 50% of their initial height. The texture attributes analyzed were hardness and fracturability. Hardness is defined as the peak force (N) recorded during the first compression cycle, while fracturability referred to the force (N) required to break the sample. The measurement was conducted at room temperature ($25^{{}^{\circ}}C\pm 2^{{}^{\circ}}C$).

### 2.5 Bulk Density and Expansion Rate

The bulk density of crackers was determined using the beads displacement method, commonly employed for irregularly shaped objects. This method utilized a known volume of uniformly sized glass beads as the displacement medium. Bulk density was calculated using the formula:

$$\text{Bulk density}=\frac{\text{Weight of sample}\;\left(g\right)}{\text{Volume of displaced beads}\;\left({\mathrm{cm}}^{3}\right)}$$

The expansion ratio of crackers was determined by comparing the volume of the raw and fried crackers. The expansion ratio was calculated using the formula:

$$\text{Expansion ratio}\;\left(\%\right)=\frac{V_{\text{fried kerupuk}}-V_{\mathrm{raw}\;\text{kerupuk}}}{V_{\mathrm{raw}\;\text{kerupuk}}}\times 100$$

### 2.6 Proximate Analysis

The proximate analysis of the crackers was conducted according to established standard methods of the Association of Official Analytical Chemists (AOAC 2005). The parameters analyzed included moisture, ash, protein, fat, and carbohydrate content. Moisture content was determined using the gravimetric method, which determined by oven drying the sample in an oven at 105°C until a constant weight was achieved (AOAC 925.10). Ash content was determined by incinerating the sample in a muffle furnace at 600°C for 6 h. The residue left was considered as the content (AOAC 923.03). Protein content was determined by the Kjeldahl method (AOAC 991.20), which involves three steps: acid digestion, neutralization and distillation. The protein content was calculated using a conversion factor of 6.25. Fat content was analyzed using the Soxhlet extraction method (AOAC, 920.39), with hexane as the solvent to isolate fats. Carbohydrate content was determined by the difference method, calculated by subtracting the sum of moisture, ash, protein, and fat content from 100%.

### 2.7 pH and Water Activity (*a*w)

The pH value of the crackers was determined by homogenizing 10% of finely ground sample with several drops of distilled water. The pH of the solution was then measured using a calibrated digital pH meter (Eutech pH 700, Singapore). The *a*w of crackers was measured using an advanced water activity meter (Aqualab 4TE, USA). The ground sample was placed into a specific sample cup. Once the *a*w value stabilized, the device emitted an audible signal indicating the completion of the measurement.

### 2.8 SEM

The microstructural characteristics of crackers were observed using a scanning electron microscope (JSM 6510, JEOL, Japan). This analysis aimed to evaluate the structural changes induced by different frying methods at the surface and cross‐section. To prepare the samples, the fried crackers were fractured into small, uniform pieces. The samples were mounted on an aluminum stub using conductive adhesive tape. The samples were then coated with a thin layer of a gold–palladium alloy using a sputter coater under a vacuum environment. The micrographs were captured at 250–1000× magnifications.

### 2.9 FTIR

FTIR analysis was conducted using a FTIR spectrophotometer (Bruker Tensor II, Germany), covering the infrared spectral range of 400–4000 cm^−1^. The obtained spectra were analyzed by comparing the absorption peaks of different samples, facilitating the identification of structural alterations due to varying frying methods.

### 2.10 Sensory Evaluation

Sensory evaluation of crackers was conducted using a 9‐point hedonic test involving 100 untrained panelists to assess the acceptance levels regarding the sensory attributes of the crackers, including texture, taste, color, aroma, and overall liking. The samples were coded with random three‐digit numbers and presented monadically to prevent direct comparison between samples. Subjects were provided with plain water to cleanse their palates between sample tastings with a 1 min break between each cracker evaluation. The detailed description of the hedonic scale was as follows: (1) Extremely dislike; (2) Strongly dislike; (3) Dislike; (4) Slightly dislike; (5) Neutral; (6) Slightly like; (7) Like; (8) Strongly like; (9) Extremely like. Finally, subjects were asked to rate the intensity of each evaluated attribute.

Before the tests began, the study introduction was given to the participants, and the digital consent forms were obtained after respondents ticked the “agreed” box in the online questionnaire to indicate their willingness to participate. Moreover, the researchers asked for verbal consent at the beginning of the evaluation, and all participation was conducted voluntarily. At the start of the test, the researchers provided the list of ingredients used in the samples to the consumers to prevent the recruitment of individuals with food allergies or dietary restrictions related to any of the ingredients. The participants were notified that all collected data and information would be processed and stored anonymously. The Research and Technology Transfer Office, Bina Nusantara University, Jakarta, Indonesia (referral code: 085/VRRTT/V/2025), approved the study, which followed the World Medical Association (WMA) Declaration of Helsinki (WMA 2013).

### 2.11 Data Analysis

The data were analyzed according to the One‐Way Analysis of Variance (ANOVA) and further complemented by Tukey's Honest Significant Difference (HSD) Post Hoc test to evaluate significant differences at a 95% confidence level $\left(\alpha =0.05\right)$. All statistical analyses were performed using IBM SPSS Statistics version 25. All the data are openly available in a public repository that issues datasets with DOIs https://doi.org/10.6084/m9.figshare.28794878.v1.

## 3 Results and Discussion

### 3.1 Determination of Selected EWP Level

To achieve the desired level of EWP, as a protein‐source food product, a preliminary test was conducted by substituting the base ingredients of crackers with varying concentrations of EWP (0%, 10%, 20%, 30%, and 40%). The results presented in Table 2 indicate that increasing the concentration of egg white protein (EWP) significantly influenced the physicochemical properties of crackers. The protein content of crackers increased significantly (*p* < 0.05) with higher levels of EWP, demonstrating its effectiveness as a high‐protein ingredient in crackers formulations. Color analysis showed that the lightness (L*) value increased with higher EWP concentrations, attributed to the naturally white color of EWP (Ekafitri et al. 2016). In contrast, the redness (a*) and yellowness (b*) values decreased with increasing EWP concentrations, suggesting a reduction in Maillard browning reactions due to the lower carbohydrate content (Starowicz and Zieliński 2019). Texture analysis showed that crackers' hardness increased with higher EWP concentrations, likely due to the formation of a dense protein matrix that restricted expansion during frying (Pakpahan and Nelinda 2019). Considering the result of enhanced protein content and color acceptability, and desirable texture, a 20% EWP substitution was identified as the optimal formulation in this study. Furthermore, this formulation has met the minimum standards required to be claimed as a source of protein, in accordance with the Regulation of the Indonesian Food and Drug Authority (BPOM) Number 1 of 2022 on Control of Claim on Processed Food Labels and Advertisements.

### 3.2 Proximate Composition

Proximate analysis is a method used to determine the fundamental nutritional components of food. The proximate composition includes moisture, ash, lipid, protein, and carbohydrate contents (Ahmed et al. 2022). In this study, proximate analysis was employed to evaluate the nutritional composition of crackers as affected by different alternative frying methods (air frying, sand frying, and microwave frying) comparing with the common method (deep frying with palm oil) as control, as presented in Table 3.

**TABLE 3:** Chemical characteristics of crackers with alternative frying methods.

Sample	Proximate						pH	*a*_w_
	Moisture (%)	Ash (%)	Protein (%)	Fat (%)	Carbohy‐drate (%)	Energy (kkal/g)		
Deep frying	4.36 ± 0.08^b^	4.51 ± 0.04^a^	15.76 ± 0.31^a^	16.55 ± 0.49^b^	58.83 ± 0.07^a^	447.31 ± 2.93^b^	7.34 ± 0.06^b^	0.42 ± 0.01^a^
Air frying	3.75 ± 0.04^a^	5.35 ± 0.01^d^	18.62 ± 0.36^b^	0.19 ± 0.01^a^	72.10 ± 0.40^b^	364.55 ± 0.17^a^	7.25 ± 0.04^a^	0.41 ± 0.01^a^
Sand frying	4.71 ± 0.06^c^	4.83 ± 0.04^b^	18.26 ± 0.14^b^	0.41 ± 0.01^a^	71.80 ± 0.23^b^	363.89 ± 0.40^a^	7.20 ± 0.04^a^	0.42 ± 0.001^a^
Microwaving	3.91 ± 0.03^a^	5.19 ± 0.04^c^	18.74 ± 0.28^b^	0.39 ± 0.01^a^	71.78 ± 0.35^b^	365.5 ± 0.32^a^	7.22 ± 0.01^a^	0.41 ± 0.004^a^

*Note:* Different superscript letters indicate significant differences at a 5% significance level (*p* < 0.05).

Moisture content is a critical characteristic of crackers, as it significantly influences the product's appearance, texture, taste, and shelf life. During the frying process, mass and energy transfer occur from the heating medium to the product, resulting in cooking and the evaporation of moisture (Nadia et al. 2023). As presented in Table 3, crackers processed using alternative frying methods (air frying, sand frying, and microwave) met the quality standard requirements for traditional crackers, with a maximum moisture content of 8%, as specified by the National Standardization Agency of Indonesia (SNI 1996). Furthermore, they showed significantly different moisture contents (*p* < 0.05) compared to the control (deep frying). These differences can be attributed to the variations in cooking temperature and duration associated with each method. Air frying required a longer cooking time due to its lower heat transfer coefficient, resulting in greater moisture loss through evaporation (Zaghi et al. 2019). Similarly, the microwave method yielded crackers with relatively low moisture content. During microwave processing, electromagnetic waves interact with water molecules within the sample, generating heat. Microwave application enhanced the dehydration rate in food materials, thereby reducing the frying time needed to achieve moisture levels comparable to those of deep frying (Sansano et al. 2018). In contrast, sand frying produced crackers with higher moisture content. This might be due to the granular nature of sand, which provides a smaller contact area between the hot surface and the sample compared to oil or air media, resulting in less efficient heat transfer and dehydration (Nirwana et al. 2018).

Ash content represents the total mineral content in food, including elements such as calcium, potassium, sodium, iron, manganese, magnesium, and iodine (Rosiani et al. 2015). The results of the ash content analysis are shown in Table 3. Alternative frying methods, namely air frying, sand frying, and microwave frying, produced crackers with significantly higher ash content (*p* < 0.05) compared to the control (deep frying). The variation in ash content among the cracker samples might be attributed to the greater reduction in moisture content associated with these alternative frying methods (Sundari et al. 2015). These findings are in line with a previous study that higher ash content in products processed using alternative frying techniques was likely due to more efficient moisture reduction, resulting in a higher concentration of minerals (Turp 2016). Furthermore, there is a potential for mineral absorption during sand frying, possibly originating from the sand medium itself. The sand puffing process can slightly increase the levels of mineral elements such as potassium, magnesium, manganese, and phosphorus (Kora 2019).

The protein content of crackers is presented in Table 3. Statistical analysis revealed a significant difference (*p* < 0.05) in protein content between crackers processed using alternative frying methods and the control (deep frying). The lower protein content observed in crackers produced using the conventional deep‐frying method might be attributed to the high level of oil absorption during the frying process. Increased oil uptake elevated fat content, which proportionally reduced the relative protein concentration. Deep‐frying reduced protein content due to oil replacing the evaporated water during frying, thereby decreasing the protein concentration per unit weight of the product (Oke et al. 2018). Another study reported similar findings, showing that replacing 10% of the base flour with rebon shrimp flour and applying deep‐frying produced crackers containing 9.03% protein (Multazam et al. 2023). Crackers produced in this study fulfilled the criteria for a “source of protein” claim as defined by BPOM, which requires that the protein content be at least 20% of the NRVper 100 g of solid food, or equivalent to 12% protein content per 100 g of solid food.

The results of the fat content analysis of crackers are presented in Table 3. Statistical analysis showed a significant difference (*p* < 0.05) in the fat content of crackers processed using alternative frying methods compared to the control (deep frying). In conventional deep frying, it was attributed to the use of oil as the heating medium. During frying, oil was absorbed into the product, resulting in increased fat levels (Pankaj and Keener 2017). Crackers were able to absorb approximately 18% of oil during the frying process (Faisal et al. 2024). The frying process caused the moisture content to evaporate due to heating. The resulting steam increased internal pressure within the crackers' pores, preventing oil from immediately penetrating during frying. Once frying was finished, the internal temperature decreased, leading to steam condensation on the surface. This created a pressure differential as a driving force that drew oil into the pores of the sample (Teruel et al. 2015). On the other hand, alternative frying methods such as air frying, sand frying, and microwave heating utilized non‐oil heat transfer media. These methods allowed samples to expand without significantly increasing their fat content. Furthermore, all of the crackers' samples in this work, produced with alternative frying methods, have met the criteria for a “fat‐free” claim as regulated by the BPOM, which required that fat content not exceed 0.5 g per 100 g. Another study reported similar findings, showing that alternative microwave cooking for 60 s produced crackers with a low‐fat content ranging from 0.25% to 0.44% (Rosiani et al. 2015).

The carbohydrate content and total energy of the crackers is shown in Table 3. Statistical analysis indicated a significant difference (*p* < 0.05) between samples processed using alternative frying methods and the control (deep frying). The difference in carbohydrate content was mainly attributed to the lower fat content in crackers prepared using the alternative method. Variations in carbohydrate content are influenced by other nutritional components, such as protein, fat, moisture, and ash (Jumiati et al. 2021). The carbohydrate content in the cracker‐based product was associated with starchy ingredients such as tapioca and wheat flour (Costa and Manihuruk 2021). Moreover, the total energy of a food product is determined by its carbohydrate, protein, and fat composition in which crackers with alternative frying exhibited significantly lower energy compared to the control (deep frying). This reduction was attributed to the higher fat content. The higher the fat content, the greater the energy contained (Pradnyani and Muflih 2024). This result was consistent with previous findings that cracker‐based products fried using oil methods had the highest energy content, whereas those processed using alternative frying methods had significantly lower energy levels. During the frying process, oil is absorbed into the crackers, substantially increasing their total energy. Fat provides a high energy value of 9 kcal/g, while carbohydrates and proteins respectively contribute only 4 kcal/g.

### 3.3 pH and *a*_w_

The pH analysis results presented in Table 3 exhibited a significant difference (*p* < 0.05) in the pH values of crackers fried using alternative methods compared to the control (deep frying). The higher pH observed in conventionally deep‐fried samples might be attributed to greater oil absorption during the frying process. It was in line with the result of the proximate analysis, discussed in an earlier section. An earlier report stated that fat oxidation and moisture loss during frying could contribute to an increase in pH (Cheng et al. 2024).

The *a*_w_ value is important for crackers and cracker‐based products, indicating their stability and crispness by determining the amount of free water available for microbial growth and texture changes. The *a*_w_ results (Table 3) indicated no significant difference (*p* > 0.05) among the samples. The high cooking temperatures involved in each method likely promoted the evaporation of free water, leading to a reduction in the final product's *a*_w_. Most microorganisms were not able to grow at *a*_w_ values below 0.60, suggesting that the crackers developed in this study were less prone to microbial spoilage (Parikh and Takhar 2016). Surprisingly, although moisture content varied significantly across treatments, *a*_w_ values remained statistically unchanged. This inconsistency might be due to differences in the bound water content within the food matrix. Bound water, which was associated with components such as, proteins, and carbohydrates, did not contribute directly to *a*_w_. Previous work investigated that heat treatment caused the release of water from cells due to the disruption of starch granules, thereby altering moisture content without substantially affecting *a*_w_ (Utama et al. 2019).

### 3.4 Color

The color characteristics of crackers are presented in Table 4. The results indicated a significant difference (*p* < 0.05) in color attributes among the alternative frying methods compared to the deep frying method. The color parameters (L*, a*, and b*) of crackers were primarily influenced by the *Maillard* reaction, a non‐enzymatic browning reaction facilitated by reducing sugars and proteins that occurred at temperatures above 120°C (Kora 2019). This reaction leads to the formation of melanoidin pigments, resulting in darker coloration (Starowicz and Zieliński 2019). Elevated temperatures and extended frying durations accelerated the *Maillard* reaction, thereby reducing lightness while increasing redness and yellowness.

**TABLE 4:** Physical characteristics of crackers with alternative frying methods.

Sample	Color				Bulk density (g/mL)	Expansion rate (%)	Texture	
	L*	a*	b*	∆*E*			Hardness (g)	Fracturability (mm)
Deep frying	90.26 ± 0.28^c^	4.16 ± 0.04^b^	30.68 ± 0.24^c^	—	0.12 ± 0.06^b^	91.09 ± 3.13^c^	1907.31 ± 27.32^c^	34.18 ± 1.14^ab^
Air frying	85.12 ± 0.12^a^	5.44 ± 0.28^c^	31.49 ± 1.51^c^	5.47 ± 0.47^b^	0.15 ± 0.05^c^	38.77 ± 1.84^a^	2552.04 ± 50.34^d^	32.89 ± 0.08^a^
Sand frying	88.19 ± 0.24^b^	4.54 ± 0.10^b^	27.55 ± 0.26^b^	3.80 ± 0.24^a^	0.11 ± 0.06^b^	55.06 ± 7.74^b^	1539.86 ± 49.52^b^	35.75 ± 0.14^b^
Micro‐waving	92.88 ± 0.23^d^	3.29 ± 0.25^a^	24.15 ± 0.36^a^	7.09 ± 0.58^c^	0.09 ± 0.003^a^	98.76 ± 7.80^c^	1312.30 ± 49.48^a^	37.58 ± 0.41^c^

*Note:* Different superscript letters indicate significant differences at a 5% significance level (*p* < 0.05).

**FIGURE 1:**
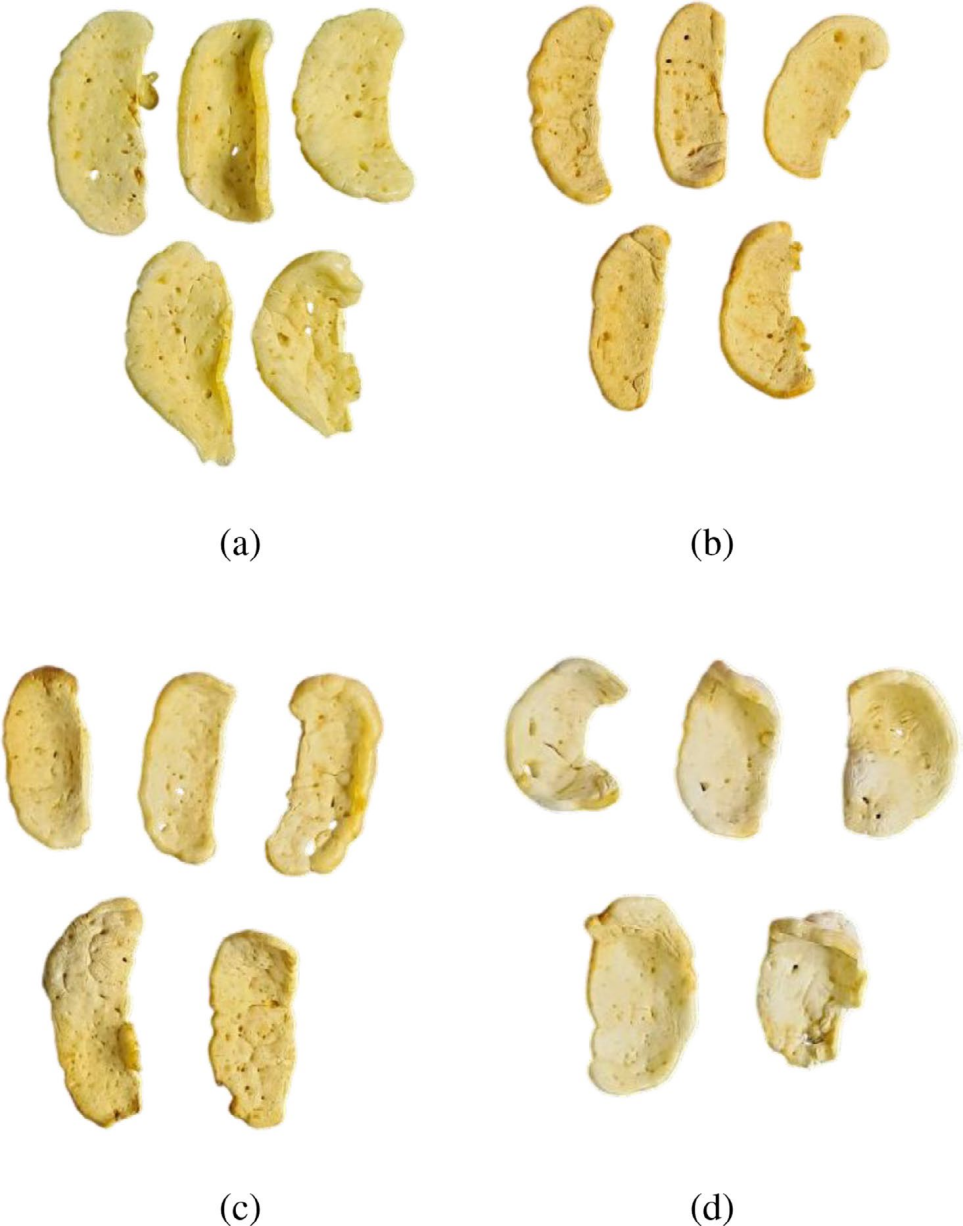
Crackers with different frying methods: (a) Deep frying (control); (b) air frying; (c) sand frying; (d) microwaving.

Microwaving produced crackers with the brightest appearance (highest L*) and minimal browning (lowest a* and b* values), likely due to limited *Maillard* reactions under electromagnetic heating conditions (Tamsir et al. 2021). Conversely, air frying produced a darker product (lowest L* and higher a* and b* values), attributed to the longer frying time that enhanced melanoidin formation (Teruel et al. 2015). Crackers processed by deep frying exhibited the highest b* value (30.68), possibly due to oil absorption creating a golden hue on the product's surface. The visual appearance of developed crackers prepared using the various frying methods is shown in Figure 1.

### 3.5 Bulk Density and Expansion Rate

The expansion rate of crackers is an important quality parameter, as greater expansion is associated with a crispier texture. The analysis results (Table 4) revealed significant differences (*p* < 0.05) in both bulk density and expansion rate among the different frying methods. Microwaving yielded the highest expansion and the lowest bulk density, whereas air frying was the opposite. These findings indicated an inverse relationship between expansion capacity and bulk density in crackers.

The variation in expansion rates was attributed to differences in heating mechanisms. Microwaving employed volumetric heating, which enabled rapid internal heat absorption and steam generation, creating high internal pressure to enhance puffing efficiency (Anggraeni et al. 2020). In contrast, air frying resulted in the lowest expansion due to slower heat transfer and delayed water evaporation, which produced lower internal pressure (Tian et al. 2017). Similarly, sand frying exhibited relatively low expansion, possibly due to limited heat transfer from the solid medium, reducing effective surface contact with the sample (Guttifera et al. 2022).

### 3.6 Texture

The texture profile of crackers is presented in Table 4. Texture analysis revealed significant differences (*p* < 0.05) in the hardness among the samples. Air frying produced the hardest product, whereas microwave frying resulted in the softest. The crispiness of crackers was indicated by the fracturability values, with microwave‐fried crackers exhibiting the most fracture samples. The expansion volume and hardness of crackers were correlated, such that during the frying process, textural changes occurred as a consequence of the interaction between the surface of the material and the heating medium (Guttifera et al. 2022). Heating induced moisture evaporation, forming air cavities and displacing water from the sample. This process led to the expansion of crackers, which directly influenced their texture (Irmayanti Syam and Jamaluddin 2017). These findings were consistent with the correlation between expansion capacity and textural properties, where greater expansion typically resulted in increased fracturability and decreased hardness (Guttifera et al. 2022). Microwave frying promoted maximal expansion, yielding a porous structure with thinner cell walls. In contrast, air frying limited expansion, producing denser crackers with increased hardness. Overall, the results demonstrate that a greater expansion volume contributes to a crispier cracker texture. Earlier investigations documented that a higher density enhanced material compactness, thereby increasing hardness and reducing the formation of cracks (Pakpahan and Nelinda 2019). Conversely, greater expansion reduced pore wall thickness, which in turn enhanced product crispiness. These phenomena were confirmed and further discussed in the next section of SEM analysis.

### 3.7 SEM

**FIGURE 2:**
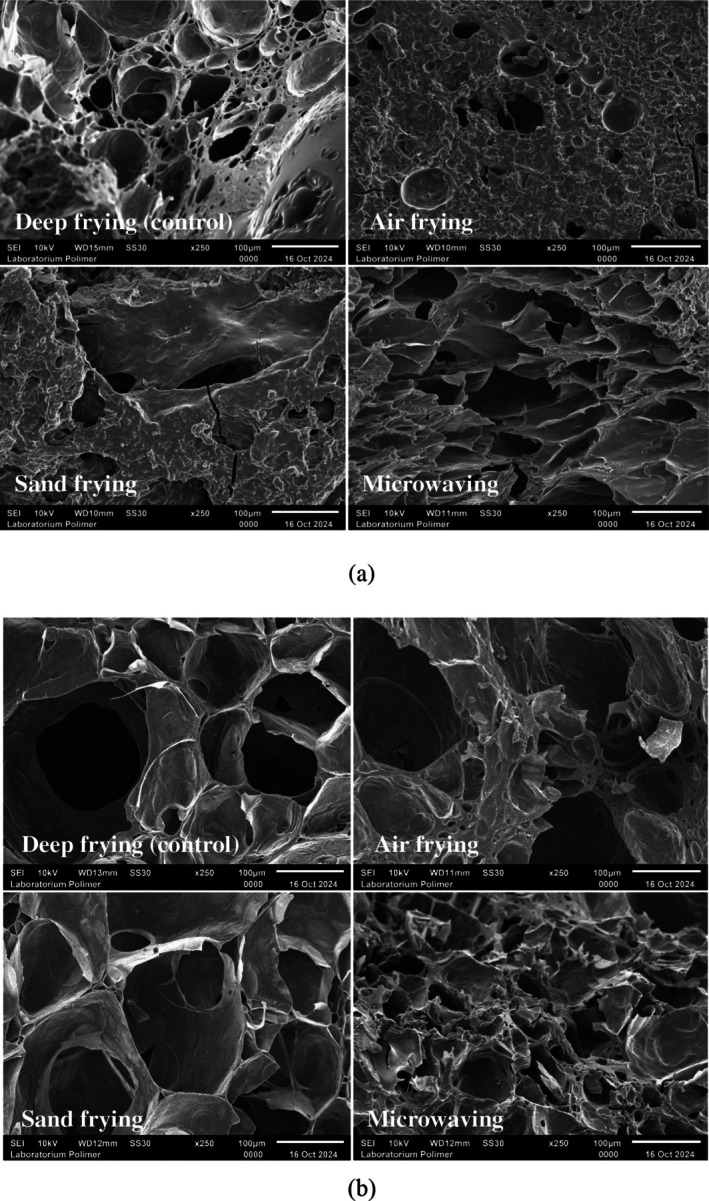
Surface (a) and cross‐section (b) microstructure of crackers.

The SEM analysis (Figure 2) presented that each frying method produced crackers with distinct microstructures. Porosity in crackers is strongly related to expansion volume and texture. Both deep‐fried and microwave‐fried crackers displayed porous structures with relatively large pores. Larger pore size indicated greater crispiness in fried products, resulting from increased expansion that led to a more hollow structure (Udokun et al. 2020). Microwaving involved the interaction of microwaves with water molecules within the sample, causing a rapid temperature rise. This generated internal water vapor pressure that moved from the core to the surface, thereby forming a more porous outer structure (Parikh and Takhar 2016).

Crackers processed by air frying and sand frying exhibited denser surfaces with fewer and smaller pores. These observations were consistent with texture analysis results, which showed that air‐fried crackers had the hardest texture due to their compact structure. Another investigation reported that air frying tended to produce products with compact and uniform structures (Wang et al. 2021). Additionally, a notable drawback of sand frying was its uneven heat distribution, which was attributed to the physical properties of sand as discrete solid particles. This limitation leads to suboptimal cracker expansion and contributes to a denser final structure (Pakpahan and Nelinda 2019).

### 3.8 FTIR

**FIGURE 3:**
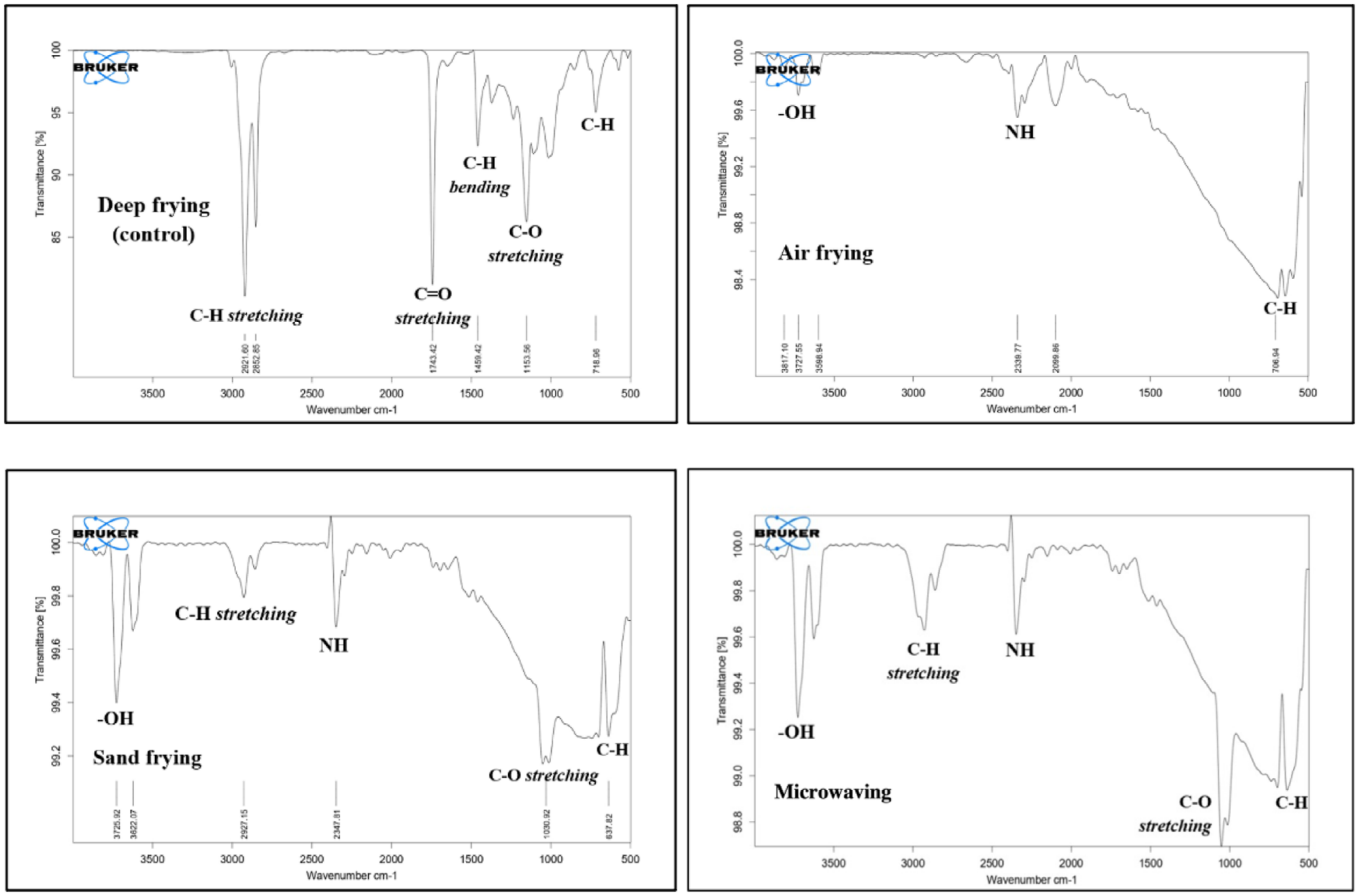
FTIR spectra of crackers.

FTIR analysis of crackers revealed the presence of several functional groups indicative of their constituent components, as seen in Figure 3. A broad absorption band observed at 3750–3600 cm^−1^ corresponded to free ‐OH (hydroxyl) groups, associated with residual moisture in the cracker samples (Figure 3) (Jozanikohan and Abarghooei 2022). Peaks in the range of 2930–2850 cm^−1^ were attributed to C‐H stretching vibrations, specifically asymmetric (2920 cm^−1^) and symmetric (2850 cm^−1^) CH_2_ bonds, indicating the presence of aliphatic organic compounds such as lipids or oils. The most intense peak was detected in deep‐fried crackers (Figure 3), accompanied by prominent C = O ester carbonyl stretching (1743 cm^−1^) and C–H bending vibrations from methylene groups (1459.42 cm^−1^), which are indicative of oil absorption during frying. Conversely, crackers processed using alternative frying methods exhibited lower lipid‐associated peak intensities, likely due to reduced direct oil uptake (Pielesz et al. 2023). Additional peaks at 2347.81–2339.77 cm^−1^ were assigned to NH stretching, suggesting the presence of amino acid groups in proteins. C–O stretching vibrations were observed in the range of 1153.56–1030.92 cm^−1^, commonly associated with ether linkages in polysaccharides, which can be attributed to tapioca flour used as the primary raw material. Furthermore, peaks at 718.96–637.82 cm^−1^ were associated with C–H bending vibrations, implying the presence of aromatic rings derived from protein‐based compounds. The frying process may also have initiated Maillard reactions, leading to the formation of aromatic compounds (Nandiyanto et al. 2019).

### 3.9 Sensory Evaluation

The sensory test was conducted to evaluate consumer acceptance of the developed crackers. The results (Table 5) showed that crackers processed using air frying and sand frying methods had significantly different overall acceptance (*p* < 0.05), while microwaved crackers did not differ significantly (*p* > 0.05) in overall acceptance from the control (deep frying). Consumer preference for crackers was primarily influenced by the acceptance of their color and texture. Crackers prepared using air frying and sand frying methods received lower scores for both attributes. These findings were consistent with the color and texture analysis presented in Table 4, which demonstrated that crackers processed by air frying and sand frying exhibited darker coloration and a firmer texture profile, thereby reducing consumer preference (Basuny and Oatibi 2016). Hence, the sample fried using a microwave emerged as the most preferred healthy cracker alternative among consumers. Furthermore, the microwaved sample showed desirable proximate properties, particularly in protein and fat content, and achieved the highest expansion rate, optimal texture with maximum fracturability, minimum hardness, and the brightest appearance.

**TABLE 5:** Sensory evaluation of crackers.

Sample	Sensory attributes				
	Color	Aroma	Taste	Texture	Overall liking
Deep frying	7.08 ± 1.47^c^	7.01 ± 1.62^b^	7.18 ± 1.54^b^	6.21 ± 2.01^c^	6.92 ± 1.41^b^
Air frying	5.64 ± 1.96^a^	5.65 ± 1.82^a^	5.48 ± 2.09^a^	4.18 ± 2.30^a^	5.14 ± 1.77^a^
Sand frying	6.12 ± 1.71^ab^	5.77 ± 1.88^a^	5.72 ± 2.04^a^	5.08 ± 2.17^b^	5.60 ± 1.77^a^
Microwaving	6.52 ± 1.73^bc^	5.86 ± 1.76^a^	6.06 ± 2.04^a^	6.75 ± 2.11^c^	6.32 ± 1.76^b^

*Note:* Different superscript letters in the table indicate significant differences at a 5% significance level (*p* < 0.05).

## 4 Conclusion

In this study, to develop protein‐source crackers, the EWP was incorporated at a concentration of 20%. Alternative frying methods, including air frying, sand frying, and microwave frying, were found to influence the characteristics of the resulting crackers, which exhibited a moisture content of 3.75%–4.71%, ash content of 4.51%–5.35%, protein content of 18.26%–18.74%, fat content of 0.19%–0.41%, and carbohydrate content of 71.78%–72.10%. Based on BPOM standards, the product met the criteria to be labeled as a source of protein and a fat‐free food. SEM analysis revealed that both microwave cooking and deep‐frying produced highly porous structures with large pores, which are associated with improved crispness. FTIR analysis indicated lower oil uptake in alternative frying methods, as demonstrated by the reduced intensities of lipid‐associated absorption bands 2930–2850 cm^−1^ (C–H stretching) and 1743 cm^−1^ (C = O ester carbonyl stretching) compared to deep‐fried samples. Among the methods tested, microwave frying produced the most favorable results, yielding crackers with the highest expansion rate (185.63%), greatest fracturability (37.58 mm), highest brightness (L* = 92.88), and organoleptic properties that were not significantly different from those of conventional crackers.

## Author Contributions

**Ata Aditya Wardana:** funding acquisition (equal), methodology (equal), supervision (equal), writing – original draft (equal), writing – review and editing (equal), **Kaneth:** conceptualization (equal), data curation (equal), investigation (equal), writing – original draft (equal). **R. Haryo Bimo Setiarto:** data curation (equal), formal analysis (equal). **Retno Wulandari:** formal analysis (equal), investigation (equal). **Laras Putri Wigati:** methodology (equal), validation (equal), writing – review and editing (equal). **Fumina Tanaka:** Formal analysis (equal); Resources (equal); Supervision (equal); Software (equal); Writing – Original Draft Preparation (equal); Writing – Review & Editing (equal). **Fumihiko Tanaka:** Formal analysis (equal); Resources (equal); Supervision (equal); Software (equal); Writing – Original Draft Preparation (equal); Writing – Review & Editing (equal).

## Consent

The authors have nothing to report.

## Conflicts of Interest

The authors declare no conflicts of interest.

## Data Availability

Data openly available in a public repository that issues datasets with DOIs https://doi.org/10.6084/m9.figshare.28794878.v1.
